# The RECAPACITA PROJECT: comparative study of the clinical, neuropsychological, and functional profile of people with severe mental disorder and partial and total capacity modification

**DOI:** 10.1007/s00127-025-02907-2

**Published:** 2025-05-26

**Authors:** Silvia Marcó-García, Georgina Guilera, Marta Ferrer-Quintero, Susana Ochoa, Gemma Escuder-Romeva, Elena Rubio-Abadal, Arantxa Martínez-Mondejar, Núria del Cacho, Vanessa Montalbán-Roca, Ana Escanilla-Casal, Sol Balsells-Mejía, Elena Huerta-Ramos

**Affiliations:** 1 Parc Sanitari Sant Joan de Déu, Barcelona, Spain; 2 Etiopathogenesis and Treatment of Severe Mental Disorders (MERITT), Sant Joan de Déu Research Institute, Sant Joan de Déu Foundation, Barcelona, Spain; 3 Department of Social Psychology and Quantitative Psychology, Faculty of Psychology, University of Barcelona, 08035 Barcelona, Spain; 4 Group of Invariance Studies for the Measurement and Analysis of Change in the Social and Health Environments (GEIMAC), Institute of Neurosciences (UBNeuro), University of Barcelona, 08035 Barcelona, Spain; 5 Biomedical Research in the Mental Health Network (CIBERSAM), Carlos III Health Institute, Madrid, Spain; 6 Germà Tomàs Canet Foundation, Sant Boi de Llobregat, Spain; 7 Research Promotion and Management Department, Statistical Support, Hospital Sant Joan de Déu (HSJD), Barcelona, Spain; 8 Research and Development Unit, Parc Sanitari Sant Joan de Déu C, Doctor Antoni Pujades, 42, 08830 Sant Boi de Llobregat, Spain; 9 Community Rehabilitation Center of Parc Sanitari Sant Joan de Déu C, Numància 5-7-9, 08029 Barcelona, Spain

**Keywords:** Decision-making capacity, Mental disease, Schizophrenia, Mental capacity, Legislation

## Abstract

**Background:**

Evaluating the decision-making capacity of individuals with Severe Mental Disorder (SMD) is essential for compliance with the 2006 Convention on the Rights of People with Disabilities. In Spain, capacity was historically determined through judicial procedures, resulting in partial or total capacity modification (CM). The abolition of this procedure in 2021 has left a gap in addressing the needs of this population, creating challenges under the new legal framework.

**Aim:**

The RECAPACITA project studied the clinical, neuropsychological, and functional profiles of individuals with SMD and CM, focusing on differences between partial (pCM) and total (tCM) modifications.

**Methods:**

A cross-sectional study was conducted with 77 adult patients with SMD and CM (47 tCM, 30 pCM) from the Parc Sanitari Sant Joan de Déu mental health network (Spain). Sociodemographic, clinical, functional, and neuropsychological data were collected, along with an independent assessment of mental capacity.

**Results:**

Around 87% of sample had a schizophrenia spectrum disorder; pCM patients presented more substance-related and personality disorders as a secondary diagnosis. While no statistically significant differences were observed between groups, clinically, tCM group presents greater clinical alteration, lower insight, sustained attention, coding capacity, processing speed and resistance to interference compared to pCM group. tCM group had worse social functioning, and lower scores in reasoning and appreciation when assessing mental capacity.

**Conclusions:**

Individuals with tCM show greater clinical impairment and higher support needs compared to those with pCM. With the practical and legal abolition of tCM, it is essential to ensure that these individuals’ persistent challenges are adequately addressed, as their needs remain significant despite the disappearance of this legal category.

## Background

All individuals are born with legal capacity, which is the recognition granted by law to all individuals to hold rights and obligations [1]. Legal capacity is essential as it encompasses all vital areas, from the right to choose one's domicile, marry or not, to signing an employment contract or exercising the right to vote. However, exercising these rights can be a complex task if the mental capacity to make these decisions is impaired. Decision-making is a multifaceted process influenced by various factors and may be mediated by our clinical, cognitive, or functional state. This creates a significant intersection between legal frameworks, which aim to protect rights, and medical evaluations, which assess the capacity to exercise those rights.

This is particularly significant when discussing individuals with Serious Mental Disease (SMD). SMD is defined as a mental, behavioral, or emotional disorder resulting in serious functional impairment, which substantially interferes with or limits one or more major life activities [2]. Under this label, various psychopathologies such as Schizophrenia, Major Depressive Disorder, or Obsessive–Compulsive Disorder are grouped, each of them with diverse clinical and cognitive symptoms, and highly heterogeneous functionality [3]. Focusing on schizophrenia, for example, clinical alterations include positive symptoms (as delusions, hallucinations, formal thought disorder, and bizarre behaviour) [4] and negative as blunted affect, alogia, avolition, asociality, and anhedonia [5]; and cognitive deficits embrace diverse alterations in executive function, memory, attention, language and processing speed [6]. Regarding the progression, while positive symptoms tend to remit and stabilize at low levels, negative symptoms persist at moderate levels throughout the lifespan [7] and neurocognitive impairments remain below the levels observed in the general population [8]. Therefore, the capacity of these individuals to make decisions has historically been questioned.

But how is it determined whether a person with SMD has the capacity to decide? There are different methods depending on the country. In most countries, capacity is assessed in a functional manner following the criteria established in their legislations. For example, in England [9], and Ontario, Canada [10], considers that a patient can be considered capable if they can understand and retain information, use this information in a reasoning process and express a choice [11]. To determine this, an evaluation is typically conducted through a semi-structured interview by a professional team, deciding whether the individual has the capacity to decide on a specific aspect (such as medication management or voluntary hospital admission). If they lack mental capacity to decide, the team provides assistance in decision-making for their best interest.

In other countries, such as Spain, this procedure differs slightly. Until 2021, mental capacity was always determined through a judicial process. That is, a judge decides whether a person has mental capacity based on various medical reports and an individualized interview with the person. If the person is unable to make a decision, the judge decides that the person's capacity should be modified, establishing its extent from partial (inability to decide only on specific areas such as money management or health decisions) to total incapacity (all areas). Through a court sentence, a curatorship or guardianship is established, respectively, to act as their representative. However, as a result of the Convention of the Rights of persons with disabilities from the United Nations in New York in 2006 [12], Spanish law was modified and the consequences of this were the elimination of the total capacity modification (CM), and the establishment of guardian figures to guide people in their decisions without representing or substituting them [13].

This new situation allows for legal incapacitation processes to be aligned with the guidelines of the New York convention but presents a dilemma for individuals judged under the old law [14]. It is imperative to understand the profile of these individuals and compare, for example, whether individuals with total capacity modification (tCM) have more overall impairment and require a higher level of support than those with partial capacity modification (pCM). Also, re-evaluations under the new framework could result in some patients being reclassified as having partial capacity, granting them access to services previously unavailable. However, it remains unclear whether they would genuinely be able to make autonomous decisions or still require assistance. This highlights the importance of analysing the variables that influence mental capacity for decision-making and implementing support mechanisms to address these complexities effectively.

There is consensus on the influence of cognitive, psychiatric, and functional factors on decision-making capacity. For example, clinical aspects such as the severity of psychopathological symptoms and lack of insight have been related to incapacity [15]. Additionally, cognitive factors such as impaired executive functions or memory can affect decision-making capacity [16], and limited participation in social activities and socio-affective relationships [17] have clear implications in this area, and must therefore be taken into account.

In the literature, when examining samples of individuals with SMD, most studies involve the use of ad-hoc tests combined with clinical interviews to classify individuals as either having or lacking the capacity to make decisions [18–20]. However, studies exploring varying degrees of capacity modification (CM) in SMD remain scarce. When addressed, these distinctions are often based on arbitrary thresholds set by specific assessment tools [21–23]. Therefore, there is limited data available comparing the clinical, cognitive, and social profiles of individuals with total capacity modification (tCM) to those with partial modification (pCM).

In this context, the RECAPACITA project was established to study the clinical, neuropsychological, and functional profiles of people with SMD and CM to understand differences between patients with CM and those without, and to identify the most influential variables in decision-making capacity [24]. In this specific work, we focus on patients with SMD and CM, aiming to characterize a sample with total and partial CM and explore the differences between the two populations, which adds a unique perspective to the understanding of this phenomenon in legally incapacitated populations.

## Methods

### Participants

We conducted a cross-sectional study, collecting data from September 2018 to May 2022. 77 individuals affiliated with the Mental Health care network of Parc Sanitari Sant Joan de Déu (PSSJD) in Sant Boi de LLobregat (Barcelona, Spain) were included in the study. To ensure representativeness of the sample, we recruited participants from three sources: psychiatric hospitalization, including acute care, subacute care, and medium and long-term care units (individuals under involuntary admission); community settings (individuals linked to community rehabilitation services within the catchment area of PSSJD); and penitentiary services, specifically the psychiatric hospitalization units at the Brians 1 and 2 penitentiary centers (Sant Esteve Sesrovires, Barcelona, Spain). Inclusion criteria were being at least 18 years of age, having a diagnosis of SMD, and having a sentence of modification of capacity. Individuals with neurodegenerative disease were excluded.

Patients were referred to the study by their clinical referents and scheduled for an individual interview with a member of the research team. Those who agreed to participate signed an informed consent declaration. The legal guardian of each participant was informed about the aims of the study and also provided consent for participation.

Participants were selected proportionally to reflect the distribution of eligible cases across the different services, maintaining the natural composition of this subgroup. This process follows a stratified sampling approach, where individuals with a sentence of modification of capacity formed the primary stratum, and selection within this group occurred without introducing additional biases. By detailing this methodology, we ensure transparency in how the sample reflects the natural distribution of individuals with a sentence of modification of capacity across clinical and community settings.

### Assessment

The evaluation was carried out at a single time point in each of the patient's referral units. To avoid bias in the assessment, the primary evaluator was blind to the patient’s type of CM. During the visit, we collected the patient's personal and family medical history, a sociodemographic questionnaire, a comprehensive neuropsychological battery, and administered a disease awareness scale. Simultaneously, the clinical scales and medical history were completed by the reference psychiatric team and, together with the guardianship referent, the social functioning scales were completed. In addition, we collected all data on the CM, as outlined in the court sentence (i.e., reason and person responsible for the CM request, causes established by the judge, year of the CM), and on areas affected, such as administration of assets, health care, promotion of social integration, and bureaucratic procedures.

The measures were:

*Sociodemographic information* Date of birth, age, ethnicity, education, children, marital and employment status, property, cohabitation situation, and type and amount of monthly income.

The clinical and functioning measures are presented in Table 1.

**Table 1:** Clinical and functioning measures

Measure	Description
Clinical Global Impression Scale (CGI)	Provides an overall estimate of the patient's clinical state, as well as subareas for positive, negative, and depressive symptoms [25, 26]
Global Assessment of Functioning (GAF)	Scores the impact of the patient's clinical symptomatology on daily functioning, ranging from 0 to 100 [27]
Life Skills Profile Scale (LSP-39)	Evaluates five areas of social functioning: self-care, interpersonal social behavior, communication and social contact, non-personal social behavior, and autonomous living [28, 29]

The neuropsychological variables considered were those reflected in the Sitges document [30]. This document was developed through consensus of experts following the Delphi method and serves as a clinical guide for cases in which decision-making capacity must be modified. It provides an evaluation protocol that indicates the different cognitive areas to consider. The neuropsychological assessment is specified in Table 2.

**Table 2:** Measures of neuropsychological assessment

Cognitive area	Instrument	Description
Level of consciousness	Glasgow Coma Scale	Evaluates basic levels of consciousness and awareness [31]
Attention	Trail Making Test Part A; Digit Subtest (WAIS-IV)	Measures attentional focus and processing speed [32, 33]*
Gnosis	Poppelreuter Test	Assesses perceptual ability [34]
Comprehensive language	Token Test	Evaluates understanding of verbal instructions [35, 36]
Expressive language	Boston Naming Test	Measures ability to produce and name verbal items [37, 38]
Calculation	Arithmetic Subtest (WAIS-IV)	Assesses arithmetic ability [33]
Episodic memory	Spain-Complutense Verbal Learning Test (TAVEC)	Evaluates verbal memory recall [39]
Orientation to reality	Positive and Negative Syndrome Scale (PANSS)	Assesses awareness of time, place, and personal identity [40]
Constructive praxis	Cubes Subtest (WAIS-IV)	Evaluates visuospatial and motor coordination [33]
Verbal fluency	Phonological and semantic fluency tests	Measures phonological and semantic fluency [36]
Resistance to interference	Stroop Test	Assesses cognitive flexibility and inhibition [41, 42]
Abstract reasoning	Reasoning Subtest (WAIS-IV)	Evaluates problem-solving and conceptual reasoning [33]
Motivation-self-control	CGI negative and depressive symptoms subscale	Measures levels of motivation and self-regulation [25]
Spontaneous ideation	CGI positive symptoms subscale	Assesses creative and spontaneous thought generation [25]
Premorbid intelligence quotient	Vocabulary Subtest (WAIS-IV)	Estimates cognitive abilities prior to illness onset [33]

*Refers to Spanish version by de la Guía E. 2012

Additional measures are showed in Table 3. To obtain an independent measure of the mental capacity of the participants, we administered the C5 brief questionnaire during the screening visit. The C5 is a semi-structured interview that has shown validity and reliability evidence for screening mental capacity [45]. It is based on the areas evaluated by standardized tests such as the MacCAT Clinical Research [44].

**Table 3:** Additional measures of the conducted assessment

Measure	Instrument	Description
Insight	Scale of Unawareness of Mental Disorders (SUMD)	Evaluates awareness of illness, medication effects, and social consequences. Scores range from 0 to 15, with higher scores indicating greater unawareness of the illness [43]
Mental capacity	C5 Brief Questionnaire	Assesses ability to understand the objectives of the study, identify possible risks and benefits, express their reasons for participating, and considers their personal values regarding the study. Scores range from 0 to 10, with a cut-off of 7 indicating lack of capacity [44, 45]

Regarding medication, we registered the prescription of antipsychotics and benzodiazepines. Given the heterogeneity of medications in the total sample of patients, Risperidone was considered as the reference antipsychotic and Diazepam as the benzodiazepine. Using equivalence tables, antipsychotic doses were converted to Risperidone doses [46] and benzodiazepines to Diazepam doses [47, 48]. Cases where equivalence could not be established were excluded from the analysis.

### Statistical analysis

Statistical analyses were performed using the SPSS 23.0 program (Armonk, NY: IBM Corp.). Statistical significance was set at *p* < 0.05. To describe the sample, we used frequencies and percentages in the case of categorical variables, and descriptive statistics for numerical values. To compare the characteristics of the two samples, we used the Student's t-test or the Mann–Whitney U test depending on the nature and distribution of each variable. The Holm–Bonferroni correction for multiple testing was applied to adjust statistical significance.

## Results

The sample consisted of 77 individuals with SMD, 47 with total CM (tCM) and 30 with partial CM (pCM).

### Sociodemographic characteristics

Table 4 summarizes the demographic characteristics of each group. The only statistical difference between groups were regarding living situation. More than half of the sample with pCM lived alone or with their family, while 72.3% of the tCM group lived in hospitals or in assisted living facilities.

**Table 4:** Sociodemographic data of each group of the sample

Sample	Total CM (*n* = 47)	Partial CM (*n* = 30)	Statistics
Age Mean (SD)	55.28 (10.56)	48.23 (10.40)	*t*(75) = 2.870 *p* = *.*005
Years of education mean (SD)	8.32 (2.68)	8.70 (2.46)	*t*(75) = − 0.626 *p* = *.*533
Variables	*n* (%)	*n* (%)	
*Gender*			
Men	35 (74.5)	22 (73.3)	χ^2^ = 0.012 *p* = *.*912
Women	12 (25.5)	8 (26.7)	
*Ethnicity*			
Eastern Europe	1 (2.1)	-	χ^2^ = 3.838 *p* = *.*428
Rest of Europe	44 (93.6)	27 (90.0)	
Gypsy	–	1 (3.3)	
North African	1 (2.1)	2 (6.7)	
South American	1 (2.1)	–	
*Marital status*			
Single	35 (74.5)	22 (73.3)	χ^2^ = 1.019 *p* = *.*961
In a stable relationship	1 (2.1)	1 (3.3)	
Divorced	6 (12.8)	5 (16.7)	
Widow/widower	2 (4.3)	1 (3.3)	
Married or cohabiting	2 (4.3)	1 (3.3)	
Other	1 (2.1)	-	
*Offspring*			
No	38 (80.9)	21 (70.0)	*t*(75) = − 1.091 *p* = *.*279
Yes	9 (19.1)	9 (30.0)	
*State in property*			
0	32 (68.1)	21 (70.0)	*t*(74) = − 0.932 *p* = *.*354
1	14 (29.8)	8 (26.7)	
7	–	1 (3.3)	
*Living situation*			
Alone	4 (8.5)	7 (23.3)	χ^2^ = 15.393 *p* = *.*009
With parents	3 (6.4)	6 (20.0)	
Own family	1 (2.1)	3 (10.0)	
Psychiatric hospital	16 (34.0)	8 (26.7)	
Assisted living	18 (38.3)	2 (6.7)	
Other	5 (10.6)	4 (13.3)	
*Work status*			
Unemployed	-	2 (6.7)	χ^2^ = 4.761 *p* = *.*190
Protected work	1 (2.1)	1 (3.3)	
Retired	8 (17.0)	2 (6.7)	
Work disability	38 (80.9)	25 (83.3)	
*Monthly income*			
0–350€	2 (4.3)	1 (3.3)	χ^2^ = 4.838 *p* = *.*304
351€–600€	26 (55.3)	10 (33.3)	
601€–800€	8 (17.0)	6 (20.0)	
801€–1000€	5 (10.6)	3 (10.0)	
More than 1001€	6 (12.8)	9 (30.0)	
*Recruiting center*			
Psychiatric hospitalization	17 (36.2)	8 (26.7)	χ^2^ = 0.984 *p* = *.*611
Penitentiary unit	4 (8.5)	2 (6.7)	
Community rehabilitation centre	26 (55.3)	20 (66.7)	

No differences were found regarding age, gender, ethnicity, marital status and employment status. The majority had no children or assets. Both groups were similar in terms of their educational background, with most participants having completed at least 8 years of primary education.

Despite it is not significant, we found a greater proportion of pCM in people receiving assistance in community rehabilitation settings. Conversely, there were more patients with tCM in hospital admission units and in the penitentiary units. Additionally, patients with pCM had a higher average monthly income, with 50% receiving over 600€ per month and 30% receiving over 1000€ per month, whereas 55.3% of patients with tCM received less than 600€ per month.

### Diagnosis

Table 5 summarizes the distribution of principal diagnosis in each group. Most people in both groups presented a diagnosis within the schizophrenia spectrum. In both groups, the most common diagnosis was schizophrenia; followed by schizoaffective disorder bipolar type and delusional disorder in the pCM group, and schizoaffective disorder of unspecified course in the tCM group. In both groups, one individual was diagnosed with a personality disorder, with borderline disorder and schizotypal disorder representing the diagnoses of the tCM and pCM individuals, respectively.

**Table 5:** Distribution of principal diagnosis in each group

Diagnosis	Total CM (*n* = 47)	Partial CM (*n* = 30)
	n (%)	n (%)
Schizophrenia spectrum and other psychotic disorders	41 (87.2)	26 (86.7)
Schizophrenia	36 (76.6)	18 (60.0)
Non-otherwise specified psychosis	–	1 (3.3)
Delusional disorder	–	2 (6.7)
Schizoaffective disorder (non-specified course)	3 (6.4)	1 (3.3)
Schizoaffective disorder (bipolar)	2 (4.3)	3 (10.0)
Schizoaffective disorder (depressive)	–	1 (3.3)
Bipolar and related disorders	1 (2.1)	1 (3.3)
Depressive disorder	–	1 (3.3)
Other mental disorders	1 (2.1)	–
Obsessive compulsive and related disorders	2 (4.3)	–
Neurocognitive disorders	1 (2.1)	–
Neurodevelopmental disorders	–	1 (3.3)
Personality disorders	1 (2.1)	1 (3.3)

Table 6 presents the secondary diagnoses. Individuals with pCM had a higher proportion of substance-related and addictive disorders, as well as personality disorders. Conversely, neurocognitive and neurodevelopmental disorders, as well as depressive disorders, were more prevalent as secondary diagnoses in the tCM group.

**Table 6:** Distribution of secondary diagnosis in each group

	Total CM (*n* = 47)	Partial CM (*n* = 30)
	n (%)	n (%)
Neurodevelopmental disorders	1 (2.1)	–
Depressive disorder	1 (2.1)	–
Neurocognitive disorders	1 (2.1)	–
Substance abuse and dependence related disorders	11 (23.4)	9 (30.0)
Disruptive, impulse control and conduct disorders	2 (4.3)	1 (3.3)
Personality disorders*	7 (14.9)	6 (20)
Schizotypal disorder	4 (8.5)	4 (13.3)
Schizoid disorder	3 (6.4)	–
Obsessive compulsive	1 (2.1)	–
Non-specified	–	2 (6.7)

*A patient had double diagnostic of personality disorder in tCM group

### Medication

The antipsychotic and benzodiazepine dosage of each group is presented in Table 7. There are no significant differences in antipsychotic consumption, indicating that both tCM and pCM individuals take similar levels of antipsychotic medication. Regarding benzodiazepine consumption, the comparison between groups suggested that patients in the pCM may had a slightly higher daily consumption of benzodiazepines than patients with tCM, but this difference was not statistically significant.

**Table 7:** Comparison of medication dose according to CM

	Risperidone		Diazepam	
	Total CM (*n* = 37)	Partial CM (*n* = 22)	Total CM (*n* = 23)	Partial CM (*n* = 15)
Mean (SD)	8.81 (8.31)	6.51 (4.43)	13.47 (13.18)	24.66 (21.58)
U Mann–Whitney test	*U* = 368.5 (*p* = .546)* r* = .078		*U* = 116.0 (*p* = .087)* r* = .277	

### Clinical profile

Table 8 presents the results of the clinical profile for each group. Statistically significant results were not obtained in any area. However, clinically significant differences between groups are observed.

**Table 8:** Comparison of clinical variables according to each capacity group

TEST	Total CM (*n* = 47)	Partial CM (*n* = 30)*	Statistical values
	Mean (SD)	Mean (SD)	
GAF**	48.60 (7.82)	53.43 (9.42)	*t*(75) = − 2.442 *p* = .017 d = .563
*CGI*			
Total	4.83 (0.70)	4.33 (0.88)	*U* = 489.0 *p* = .014 r = .279
Negative symptoms	4.36 (1.11)	4.03 (1.18)	*U* = 594.0 *p* = .231 r = .137
Depression symptoms	1.91 (0.99)	1.97 (0.80)	*U* = 651.5 *p* = .551 r = 0.068
Positive symptoms	3.17 (1.22)	2.67 (1.06)	*U* = 527.0 *p* = .054 r = .219
*SUMD*			
TOTAL	10.15 (4.29)	7.86 (4.50)	*U* = 477.5 *p* = .028 r = .196
Awareness of having a mental disorder	3.38 (1.55)	2.69 (1.75)	*U* = 529.0 *p* = .088 r = .223
Awareness of the effects of medication	3.21 (1.70)	2.45 (1.52)	*U* = 507.5 *p* = .052 r = .255
Awareness of the social consequences of having a mental illness	3.55 (1.62)	2.66 (1.67)	*U* = 483.5 *p* = .026 r = .253

*In SUMD scale from pCM group, the scores refer to a n = 29

**In GAF test, the statistic used was Student's t test. In CGI and SUMD, the Mann–Whitney U was used

tCM group had greater clinical alteration in most of the scales administered. Individuals with tCM had worse global functioning, greater global symptomatology and higher presence of positive symptoms. They also had less awareness of their illness, the effects of medication, and the social consequences of their disorder, with a very low capacity for insight, when comparing with patients with pCM.

A categorical analysis was conducted for the CGI domains (depressive, positive, and negative), focusing on the distribution of severity levels (“Low,” “Moderate,” and “Severe”) across both groups. There were no statistically significant differences in the Depressive domain (*p* = 0.509), Negative domain (*p* = 0.270) or Positive domain (*p* = 0.100). But it is worth to point that, in the positive domain, there was a higher proportion of cases in the "Severe" category, and a slight linear trend was observed (*p* = 0.021), suggesting a potential relationship between greater clinical severity and tCM.

### Cognitive profile

The neuropsychological results obtained by each group are shown in Table 9. The scores are presented in scaled scores (they are the raw test scores corrected for age and education, with a Mean = 10, SD = 3).

**Table 9:** Comparison of scalar scores in the neuropsychological battery between people with tCM and pCM

TEST	Total CM (*n* = 47)*		Partial CM (*n* = 30)*		Statistical values
	Mean (*SD*)	CI 95%	Mean (*SD*)	CI 95%	t de Student (sig. Bilateral)
Direct digits*	7.28 (2.99)	(6.40. 8.15)	8.66 (2.97)	(7.52. 9.79)	*t*(74) = − 1.955 *p* = .054 d = .459
Inverse digits*	8.04 (2.67)	(7.26. 8.83)	8.34 (2.66)	(7.33. 9.36)	*t*(74) = − 0.480 *p* = .633 d = .113
Boston Naming Test	7.19 (2.88)	(6.34. 8.04)	7.62 (3.01)	(6.47. 8.77)	*t*(74) = − 0.619 *p* = .538 d = .145
Block design (WAIS-V)	6.85 (3.02)	(5.96. 7.74)	6.67 (3.16)	(5.48. 7.85)	*t*(75) = 0.256 *p* = .799 d = .059
Semantic fluency*	6.83 (2.64)	(5.45. 7.14)	6.45 (2.75)	(5.40. 7.50)	*t*(74) = − 0.225 *p* = .823 d = .503
Fluency (Animals)*	5.96 (2.57)	(5.20. 6.71)	6.03 (2.41)	(5.12. 6.95)	*t*(74) = − 0.868 *p* = .897 d = .030
Stroop test: word*	6.49 (2.92)	(5.63. 7.35)	6.21 (2.06)	(5.42. 6.99)	*t*(73) = 0.493 *p* = .623 d = .107
Stroop: Word-colour*	5.55 (2.54)	(4.81. 6.30)	5.71 (2.49)	(4.75. 6.68)	*t*(73) = 0.269 *p* = .789 d = 0.064
Vocabulary (WAIS-V)*	7.94 (3.68)	(6.86. 9.02)	8.79 (3.53)	(7.45. 10.14)	*t*(74) = − 1.001 *p* = .320 d = .235
Estimate of premorbid IQ**	89.68 (18.39)	(84.28. 95.08)	93.97 (17.69)	(87.23. 100.70)	*t(*74) = − 1.001 *p* = .320 d = .235
TMT-A	6.61 (2.91)	(5.74. 7.47)	6.67 (3.59)	(5.32. 8.01)	*U* = 575.0 *p* = .222 r = .140
Token Test	7.33 (4.51)	(5.98. 8.67)	7.41 (4.51)	(5.70. 9.13)	*U* = 514.0 *p* = .095 r = .192
Arithmetic subtest (WAIS-V)	6.72 (2.35)	(6.03. 7.42)	7.28 (2.23)	(6.43. 8.13)	*U* = 561.5 *p* = .193 r = .149
Matrix Reasoning (WAIS-V)	6.06 (2.85)	(5.23. 6.90)	5.79 (2.73)	(4.75. 6.83)	*U* = 667.0 *p* = .876 r = .018
Poppelreuter Test	8.98 (1.89)	(7.72. 12.55)	9.19 (1.56)	(8.62. 9.79)	*U* = 693.0 *p* = .870 r = .019
TAVEC Total codification	3.55 (3.36)	(2.56. 4.54)	5.5 (3.3)	(4.27. 6.73)	*U* = 458.5 *p* = .008 r = .307
TAVEC Short term memory	4.33 (3.42)	(3.31. 5.32)	4.99 (3.27)	(3.79. 6.22)	*U* = 631.5 *p* = .424 r = .092
TAVEC Long term memory	2.47 (3.36)	(1.48. 3.46)	3.61 (4.35)	(1.96. 5.23)	*U* = 594.0 *p* = .228 r = .138
TAVEC Long term memory (recognition)*	8.5 (5.16)	(6.97. 10.03)	8.89 (3.63)	(7.54. 10.27)	*U* = 667.5 *p* = .804 r = .028

*In the Total CM group, the N = 46 in the TMT-A, Token and TAVEC Recognition tests. In the Partial CM group, the N = 29 in all tests except in the Block design subtest (N = 30) and in the Stroop Word-Color subtest (N = 28)

**Scores from the estimated premorbid IQ are direct intelligence quotient (Mean = 100, SD: 15)

Both groups presented mild deficits in tests of memory (short and long-term encoding and recall) and executive functions (phonological fluency and resistance to interference). Both groups maintained within-normal performance in the areas of processing speed, attention, working memory, visual discrimination, language (comprehension, expression and vocabulary level), visoconstructive praxis and verbal fluency.

A subsequent subanalysis was performed to determine whether any patients demonstrated completely normal cognition, defined as achieving scaled scores above 7 on key cognitive tests (Trail Making Test, Digit Span, Matrices, Vocabulary, and others) and z-scores above -1 in TAVEC and Poppelreuter. The results revealed that no patients met these criteria.

Just as in the clinical profile, there were also no statistically significant differences in the neuropsychological profile between individuals with different degrees of capacity modification. However, clinically, it can be observed that the tCM group presented lower sustained attention capacity, reduced processing speed and resistance to interference, as well as diminished coding capacity. Although the estimated premorbid IQ in the tCM group is lower than in pCM, this difference is not significant. Both tCM and pCM individuals have an intellectual level within the normal range before the onset of the disease.

### Social functioning profile

Table 10 presents measures of social functioning in individuals with CM. Although no statistically significant differences were observed, clinically the tCM group had lower overall functioning, as well as in four of the five subareas, with communication and social contact, and autonomous life, scoring the lowest.

**Table 10:** Comparison of the social functioning of the CM groups evaluated with LSP-39 scale

	Total CM (*n* = 47)	Partial CM (*n* = 29)	Statistical values
	Mean (SD)	Mean (SD)	
Self-care	34.43 (5.22)	34.90 (4.76)	*U* = 651.5 *p* = .747 r = .037
Interpersonal social behaviour	34.74 (4.31)	35.76 (4.24)	*U* = 572.5 *p* = .241 r = .135
Communication and social contact	19.43 (3.61)	20.55 (3.11)	*U* = 559.5 *p* = .189 r = .151
Non-personal social behaviour	22.38 (2.10)	21.69 (2.43)	*U* = 580.0 *p* = .257 r = .130
Autonomous life	16.47 (4.74)	17.97 (4.21)	*t*(74) = − 1.393 *p* = .168 d = .327
TOTAL	127.45 (14.02)	130.86 (13.65)	*t*(74) = − 1.041 *p* = .301 d = .244

The scores of the independent assessment of patients' mental capacity, evaluated with the C5 brief questionnaire, are shown in Table 11. In both groups the total score was below the cut-off point (i.e., 7), but it was lower in the tCM group. There were no statistically significant results in any area, indicating that the tCM group and pCM group had similar difficulties in recognizing the risks of the study, reasoning their participation, understanding the objectives of the study, considering its benefits, and evaluating the implication of their personal values when participating.

**Table 11:** Comparison of the direct results obtained in the brief C5 Questionnaire between both groups of CM

	Total CM (*n* = 47)	Partial CM (*n* = 29)*	Statistical values
	Mean (*SD*)	Mean (*SD*)	
Objectives of the study	0.89 (0.66)	1.17 (0.65)	*U* = 534.0 *p* = .080 r = .201
Benefits of the study	0.94 (0.60)	1.07 (0.59)	*U* = 607.0 *p* = .348 r = .108
Risks of the study	1.51 (0.62)	1.79 (0.41)	*U* = 523.5 *p* = .042 r = .234
Reason for participating	0.81 (0.64)	1.21 (0.67)	*U* = 473.0 *p* = .014 r = .283
Personal values	0.98 (0.67)	1.21 (0.72)	*U* = 561.5 *p* = .160 r = .161
Total	5.13 (2.71)	6.45 (2.45)	*U* = 466.0 *p* = .020 r = .267

*Total sample was from 29 patients due to one patient dropping out during assessment

## Discussion

This study provides data on the clinical, neuropsychological and functional profile of individuals with CM, differentiating between those who require support in all areas of their lives (tCM) and those who need support in specific areas such as economic or health management (pCM).

Traditionally most studies have evaluated capacity in a dichotomous way [15, 49]. Frequently, researchers administer the MacArthur Competence Assessment Tool (MacCAT) ad-hoc, and conduct a clinical interview, categorizing them into those with capacity to decide (treatment options or participation in clinical studies, for example) and those without [18–20]. There are very few studies that address different degrees of CM in SMD, and typically these are delineated by an arbitrary cutoff point on the MacCAT test. For instance, in Mandarelli et al.'s study in 2018, the sample was divided between high (patients scoring more than 75% in the subscales and maximum score in expressing a choice) and low capacity [23]. In the same year, another study by Murphy et al. (2018) considered cutoff points for each of the subscales, finding that only 1.7% of the sample had partial capacity, while 27.7% lacked capacity, and associations between demographic (age, marital status, employment) and clinical variables (number of diagnoses) with lack of mental capacity were discovered [22]. Interestingly, another study by Curley et al. (2019a) that graded capacity (total, partial, or lacking) found that over half of the analyzed sample (50.7%) had partial mental capacity, and that demographic (age, employment, ethnicity) and clinical (voluntary admission status) factors only explained 27.6% of the variance in mental capacity between participants [21]. In both studies, the authors proposed quantifying the role of cognitive and other factors in relation to unexplained variability.

Based on these conclusions, this study aims to provides a detailed description of the cognitive, psychiatric and functional profiles of individuals with varying degrees of capacity and offers a comparison between groups, making it specially relevant in this field. The relevance of this work lies in the fact that, statistically, there are no significant differences between patients with pCM and tCM in terms of their psychopathological, cognitive, or functional profiles. This raises an important debate, as the degree of capacity modification does not seem to depend on any of these variables, but rather may be conditioned by the combination of all of them at a specific moment. Nevertheless, clinical differences are observed, and therefore should be taken into account.

The sociodemographic profiles indicate that persons with tCM experience a higher prevalence of involuntary psychiatric admissions than persons with pCM. These results are consistent with previous studies [49, 50]. A plausible explanation is that individuals requiring involuntary hospital admission often present more severe clinical and behavioral symptomatology, posing a risk to themselves or others [51, 52]. Such symptomatology can directly impact their decision-making capacity [53], as evidenced by our results showing that patients with tCM had greater global and positive symptomatology than those with pCM, with greater severity, according to the results obtained from the categorical analysis of CGI. However, the relationship between positive symptoms and lack of capacity remains a subject of ongoing debate [16, 54]. Patients in both groups were comparable in terms of age, gender, academic background, employment status, and number of offspring, but individuals with tCM presented a lower socioeconomic status than those in the pCM group. Although most authors conclude that there is no relationship [55], associations between lower economic status and capacity have been described in some cases [15]. In Spain, the monthly income of a person with SMD and CM usually comes from government grants that consider work history (if the worker is completely unable to work, their reference salary is reduced to the minimum benefit) or additional costs associated with disability (such as constant care or home adaptations). All of these factors may necessitate a higher level of financial support to cover these expenses. Additionally, within our sample, individuals with pCM tended to reside either independently or with family members, thereby potentially reinforcing this hypothesis.

Schizophrenia was the most prevalent diagnosis in both groups, aligning with its known association with disability [56–58] and unawareness of illness [43]. The results of the present study reinforce the need to strengthen programs that aim to prevent first-episode psychosis and provide therapeutic support for the difficulties faced by individuals with tCM. Individuals with tCM presented more severe diagnoses than those with pCM, such as higher rates of diagnosis of schizophrenia, OCD, and Impulse Control Disorder, which are associated with severe clinical impairment [59–61] and severe impairment in global clinical functioning (GAF scale). Conversely, the pCM group presented a higher prevalence of substance use disorders, potentially influenced by their social environment. Specifically, patients with tCM were more often in inpatient and prison units, environments where there is usually greater control of substance use and with less access to them compared to those living independently. Moreover, this may explain why the pCM group showed a slightly higher consumption of benzodiazepines than the tCM group. The literature has also reported the relationship between higher benzodiazepine use in people with SMD and a diagnosis of substance use disorder [62] and especially in those in the community [63]. Benzodiazepine use may help reduce interpersonal conflicts and improve coexistence between the individuals, their family, and the community. Despite these clinical and environmental differences, social functioning, as measured by the LSP-39 scale, did not significantly vary between groups. This may reflect the scale’s subjective nature, especially when assessed by guardians who oversee multiple individuals (some with pCM and others with tCM). In fact, the influence of the clinical point of view in the assessment of mental ability is an issue that has been previously analyzed [64], and although there are some conflicting results [65], most consider that it does not entail biases [21].

On the other hand, it's noteworthy that both groups obtained a low score for the area of Autonomous life. This indicates that both people with tCM and pCM have difficulties to fend for themselves in their day to day. This is highly relevant since the deficit in this area is likely the primary motivator for initiating a CM process. Consequently, these results emphasize the need for a careful approach to CM, prioritizing the enhancement of individual autonomy. This can be achieved through targeted interventions such as occupational therapy or support from other professionals dedicated to fostering independence, especially when decision-making capacity is compromised. Related to this point, our results point out that the clinical evaluation of insight, as measured by the c5 scale, is consistent with the judicial evaluation: individuals with tCM have the lowest decision-making capacity, particularly in assessing the risks of the study and arguing the reasons for participation. This partially supports the results of Curley's study [49], which concluded that patients with tCM have worse reasoning ability compared to those with pCM. The SUMD, our measure of clinical insight, also yielded group differences. Clinically, people with tCM were less aware of the effects of medication, and the social consequences of their disorder than the pCM group. This was anticipated, as existing literature has established a strong relationship between insight and capacity [55, 58], and its impact on legal decisions surrounding a person's mental capacity. In line with these results, it is therefore crucial to prioritize interventions aimed at improving insight in individuals with tCM and to look for an improvement in their decision-making capacity. Previous studies [66–68] have demonstrated the efficacy of such interventions in boosting insight. Further research is needed to fully understand the impact of improved insight on other clinical variables [69, 70], but it remains a promising line of research.

At a cognitive level, the capacity for mnesic coding and executive functions (sustained attention, processing speed and resistance to interference) were shown to be key areas of differences between the two groups. Individuals with tCM performed significantly worse than those with pCM. Although there are scarce studies that have evaluated cognitive ability in patients with different degrees of capacity, the literature has reported mnesic and executive impairment in people with lack of capacity [71, 72], consistent with the results of our study. Interestingly, the observed cognitive impairment was mild (one standard deviation below the mean), both in tCM and pCM groups. This suggests that the cognitive profile of people with SMD and CM is not significantly more altered than those with SMD alone. It can be concluded therefore that capacity modification is not primarily dependent on cognitive level, but rather on the level of daily functioning or insight. Given the heterogeneity and dynamic nature of SMD, assessments of decision-making capability may inadvertently bias towards a perceived need for greater support than is actually necessary. As cognitive factors play a crucial role in the decision-making process, their assessment and the provision of appropriate support should take into account the actual limitations faced by individuals with SMD. Considering the weight that cognitive variables play in the decision-making process, their evaluation and support should be considered, taking into account the true limitations of individuals with SMD.

This work must be interpreted considering certain limitations. First, our sample contains considerable heterogeneity within subgroups, and thus some comparisons may lose representativeness. At the same time, the sample is composed by mostly patients with schizophrenia spectrum which may limit the generalizability of the findings to the broader population of individuals with severe mental disorders (SMD). Second, assessing patients already judged as incapable may generate bias in the assessment of clinical or social functioning. Third, the sampling strategy and a limited sample size prevented us from generalizing our results to a broader population. Fourth, our study does not account for disease progression or other factors that could have influenced the judicial decision regarding the type of capacity modification. In Spain, capacity modification is determined through a judicial ruling based on the evidence available at a specific point in time. Consequently, our cross-sectional design does not include variables that may have evolved after the judicial decision. Future research with longitudinal designs could provide valuable insights into how these factors influence capacity over time. Another potential limitation refers to the possibility of selection bias. Factors such as consent capacity and recruitment site (e.g., hospitalization, forensic units, or community settings) may have influenced the characteristics of the sample. While these differences reflect the real-world heterogeneity of individuals with SMD, they could limit the generalizability of the findings. Future studies could address this limitation by conducting stratified sub-analyses to explore the potential impact of these variables in greater detail. Despite these limitations, given the scarcity of data in this area, we consider our study to be a valuable starting point.

## Conclusions

This study offers a comprehensive examination of clinical, neuropsychological, and functional data among individuals with varying degrees of mental capacity. Although no statistically significant differences were found in terms of psychopathological, cognitive, or functional profiles between the two groups, clinical differences were observed and should be taken into account. Individuals with tCM are more likely to live in a hospital context, earn lower monthly incomes, have a higher rate of diagnoses with more severe symptomatology (especially positive symptoms), show worse global functioning, as well as lower awareness of illness, and demonstrate diminished memory and executive performance, compared to people with pCM. These findings suggest that capacity modification is not solely dependent on psychopathological or cognitive variables, but rather results from a complex combination of clinical and social factors at a given moment.

The evaluation of the patient's daily autonomy and insight are key aspects both in people with tCM and pCM, and their inclusion in programs to improve both aspects can help empower these people and improve their decision-making. In addition, a careful evaluation of cognitive abilities is essential for pinpointing patients' limitations in daily life, thereby facilitating targeted support where it is most needed.

Finally, these data are particularly relevant in the current legal context and can help provide an answer in the processes of assessing the capacity of people with SMD. This study underscores the importance of personalized approaches to capacity modification, with a particular focus on improving autonomy and decision-making capacity in individuals with tCM. As future steps, being able to compare the sample of people with SMD and CM with people without CM to detect possible cognitive, psychiatric and global functioning differences will pave the way for more research in the field.

## Data Availability

No datasets were generated or analysed during the current study.
